# Machine Learning-Based CT Radiomics Method for Identifying the Stage of Wilms Tumor in Children

**DOI:** 10.3389/fped.2022.873035

**Published:** 2022-05-23

**Authors:** Xiao-Hui Ma, Liqi Shu, Xuan Jia, Hai-Chun Zhou, Ting-Ting Liu, Jia-Wei Liang, Yu-shuang Ding, Min He, Qiang Shu

**Affiliations:** ^1^The Children's Hospital, Zhejiang University School of Medicine, National Clinical Research Center for Child Health, Hangzhou, China; ^2^Department of Neurology, Warren Alpert Medical School of Brown University, Providence, RI, United States; ^3^Department of Biomedical Engineering, Key Laboratory for Biomedical Engineering of Ministry of Education, School of Biomedical Engineering and Instrument Science, Zhejiang University, Hangzhou, China

**Keywords:** Wilms tumor, clinical staging, CT, radiomics, machine learning

## Abstract

**Purpose:**

To develop and validate a machine learning-based CT radiomics method for preoperatively predicting the stages (stage I and non-stage I) of Wilms tumor (WT) in pediatric patients.

**Methods:**

A total of 118 patients with WT, who underwent contrast-enhanced computed tomography (CT) scans in our center between 2014 and 2021, were studied retrospectively and divided into two groups: stage I and non-stage I disease. Patients were randomly divided into training cohorts (*n* = 94) and test cohorts (*n* = 24). A total of 1,781 radiomic features from seven feature classes were extracted from preoperative portal venous–phase images of abdominal CT. Synthetic Minority Over-Sampling Technique (SMOTE) was used to handle imbalanced datasets, followed by a *t*-test and Least Absolute Shrinkage and Selection Operator (LASSO) regularization for feature selection. Support Vector Machine (SVM) was deployed using the selected informative features to develop the predicting model. The performance of the model was evaluated according to its accuracy, sensitivity, and specificity. The receiver operating characteristic curve (ROC) and the area under the ROC curve (AUC) was also arranged to assess the model performance.

**Results:**

The SVM model was fitted with 15 radiomic features obtained by *t*-test and LASSO concerning WT staging in the training dataset and demonstrated favorable performance in the testing dataset. Cross-validated AUC on the training dataset was 0.79 with a 95 percent confidence interval (CI) of 0.773–0.815 and a coefficient of variation of 3.76%, while AUC on the test dataset was 0.81, and accuracy, sensitivity, and specificity were 0.79, 0.87, and 0.69, respectively.

**Conclusions:**

The machine learning model of SVM based on radiomic features extracted from CT images accurately predicted WT stage I and non-stage I disease in pediatric patients preoperatively, which provided a rapid and non-invasive way for investigation of WT stages.

## Introduction

Wilms tumor (WT) is the most common renal tumor and the second most common intraabdominal tumor in pediatric patients, accounting for approximately 90% of all renal neoplasms and 7% of all malignant tumors (1–3). The estimated annual incidence of WT is 7 to 10 cases per million for children younger than 15 years (4).

Prognosis and treatment of WT are closely linked to tumor risk stratification, which is based on clinical, surgical, and biological factors. Tumor stages and histological subtypes are the most important factors for treatment outcomes and survival (5, 6).

A lower stage predicts a positive prognosis as in most tumors (5, 6), despite age, tumor weight, and histological type also playing a role in WT outcome. Studies showed that patients in stage I WT, without renal capsule penetration/renal sinus infiltration and vascular metastasis, had the potential to perform nephron-sparing resection (7) or receive nephrectomy only without postoperative chemotherapy (8).

However, histological stages of WT need to be determined by en bloc resection (9). If we can develop a technique to assess WT stage I disease preoperatively, it will be helpful in the treatment decision-making and surgical individualization strategies.

Radiomics is a novel imaging technique that extracts a large number of quantitative information describing the phenotypic characteristics of lesions from CT, positron emission tomography (PET), Magnetic resonance imaging (MRI), and other medical images (10, 11). Radiomics, combined with appropriate mathematical algorithms, have demonstrated advantages in tumor histological subtypes classification (12), staging (12), distinguishing lymph node metastases (13), and prognosis analysis (14) to provide clinical decision support, hence, has a wide application prospect (15).

This research aims to use CT-based radiomic features to build a machine-learning model to discriminate WT stage I and non-stage I disease in pediatric patients before surgery.

## Methods

### Objects

A retrospective study was conducted to analyze hospitalized patients with WT from October 2014 to October 2020. This study was approved by the hospital's ethical committee (No. 2021-IRB-097).

The inclusion criteria included the following: (1) CT-enhanced abdominal scan before surgery, biopsy, radiotherapy, or chemotherapy; (2) subsequent successful tumor resection; and (3) postoperative pathology confirmed as WT. Exclusion criteria were shown as follows: (1) Contrast-enhanced CT scan of the abdomen was not carried out or treatment was performed before CT examination; (2) radiotherapy, chemotherapy, or both of them were implemented before surgery; (3) Pathology was unclear; (4) bilateral WT; and (5) Motion artifacts in CT images, which affect observation. A total of 118 WT cases were included in this study based on the above criteria.

### WT Staging

Patients with WT were divided into two groups based on the Children's Oncology Group (COG) staging criteria: stage I and non-stage I, and the preoperative assessment is the focus of this study. Stage I conditions include the following: (1) The tumor is confined to the kidney and the renal capsule is intact; (2) the tumor has not ruptured or been biopsied before surgical resection; and (3) tumor cells have not invaded the renal sinus system (1). The rest is labeled as non-stage I. There were 48 patients with stage I disease and 70 cases with non-stage I disease among the 118 patients WT, who were recruited (Supplementary Table S1).

### Image Acquisition and Analysis

#### Image Acquisition

Two types of multi-slice helical CT (Optima CT660 CT, GE Medical Systems, and Somatom Emotion 16, SIEMENS) were used for examination. The scanning parameters of GE Optima CT660 CT were tube voltage of 120 kV, tube current is 80 mA, the layer thickness of.625mm, and the field of view (FOV) is 350x350 mm, and the matrix is 512x512. The Somatom Emotion 16 CT scanner parameters were tube voltage of 110 kV, tube current of 75 mA, the layer thickness of 1.5mm with FOV of 350 × 350 mm, and matrix of 512 × 512. Non-ionic iodine contrast agent was injected intravenously with a high-pressure syringe (Mallinckrodt Injection System, Liebel-Flarsheim Co.) and was dosed according to body weight (1.5 ml/kg) at a rate of 1.5–2 ml/kg. Portal venous phase images were acquired with a delay of 50 s after the injection.

The CT images are retrieved from the Picture Archiving and Communication System (PACS) for radiomics analysis.

#### Image Processing

The retrieved portal venous phase CT images were uploaded to a secure laptop. Then, a 3-dimensional slicer (16) (3DSlicer, 4.11.0, http://www.slicer.org/) platform was used to delineate the three-dimensional (3D) regions of interest (ROIs) of renal masses with a semi-automatic segmentation procedure, which was performed by two senior radiologists who had more than 10 years of clinical experience.

Firstly, inter- and intra- observer repeatability of feature extraction was evaluated by Intra Class Correlation Coefficient (ICC) with images of 60 (17) randomly selected cases for analysis in a blind way by the two radiologists. Radiologist 1 (J. Xuan) repeated the generation of radiomic features twice within 2 weeks following the same procedure to observe the intra-observer reproducibility. Simultaneously, radiomic features were extracted by radiologist 2 (H.C. Zhou) using the same methodology with the same set of images, which were used to assess inter-observer reproducibility between radiologist 1 (J. Xuan) and radiologist 2 (H.C. Zhou). An ICC of higher than 0.75 was considered to have good agreement (17). Then, radiologist 1 (J. Xuan) completed the workflow for the rest images.

#### Feature Extraction

Pyradiomics (18), an open-source toolbox based on Python (v3.0, https://pyradiomics.readthedocs.io/en/latest/), was deployed to analyze and extract the internal imaging features of tumor ROI in patients with WT. The overall workflow is as follows:

1) All the ROI of images were resampled into isotropic data with voxel spacing of 1^*^1^*^1 mm^3^ with a B-spline algorithm, discretized into a bin width of 25, and normalized (with the normalized scale of 500) to a consistent gray value by Pyradiomics in the first place.

2) Filters (including wavelet, Laplacian of Gaussian, Exponential, Logarithm, Square, Square Root, Gradient, Local Binary Pattern 2D, and Local Binary Pattern 3D filter) were applied to transform the images. Both the original images and the derived images using filters were arranged to extract features.

3) The extracted radiomic features incorporated First Order Statistics, Shape-based 3D, Gray Level Co-occurrence Matrix (GLCM), Gray Level Run Length Matrix (GLRLM), Gray Level Size Zone Matrix (GLSZM), Neighboring Gray Tone Difference Matrix (NGTDM), and Gray Level Dependence Matrix (GLDM).

4) A total of 1,781 radiomic features were generated from the tumor ROI of each patient with WT.

### Feature Selection, Classification, and Validation

**Figure 1:**
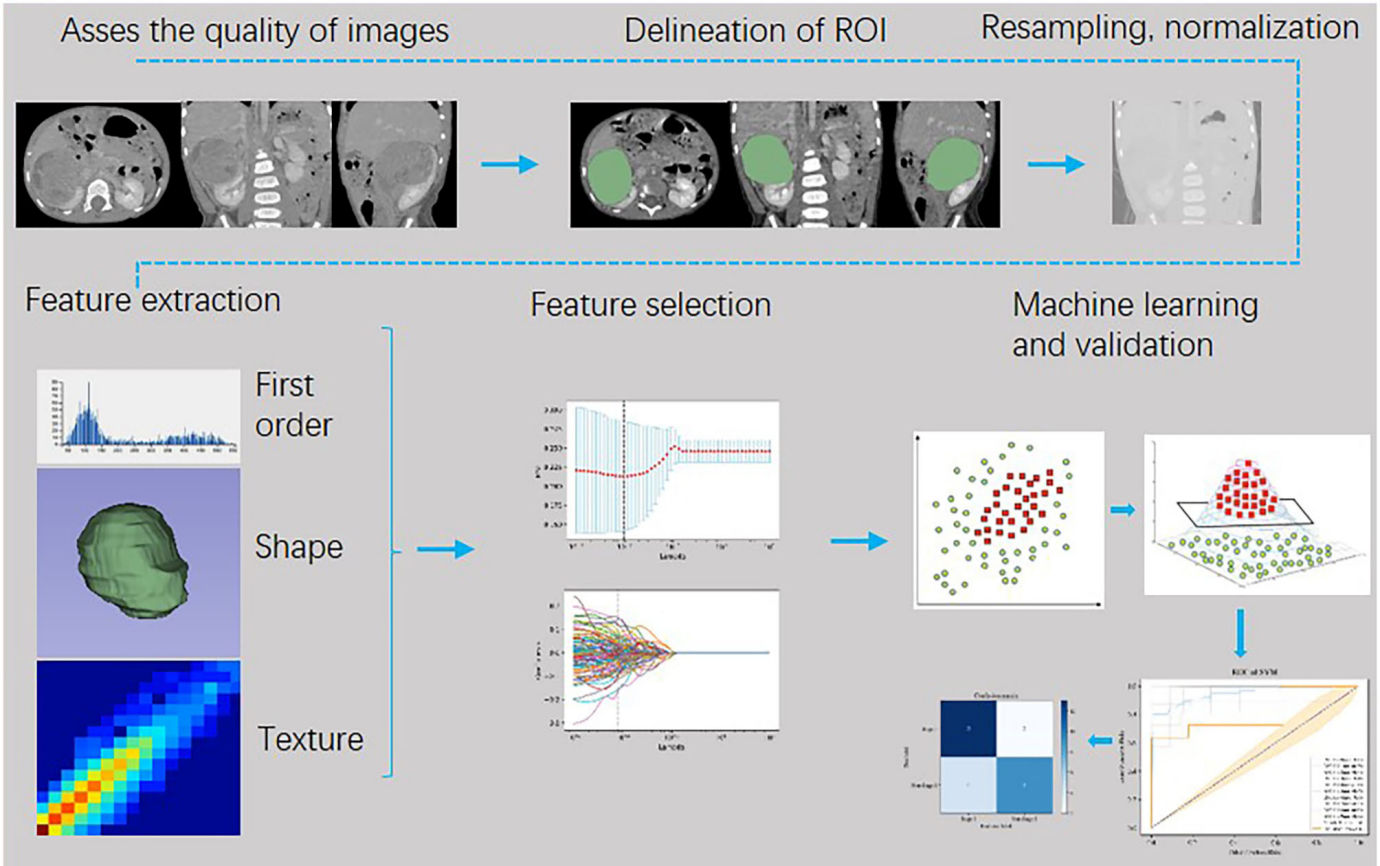
Flowchart of Wilm's tumor (WT) staging based on machine learning model of radiomics.

The workflow of image processing, radiomics feature extraction, and machine learning is shown in Figure 1. Python (Version 3.7.3, https://www.python.org) was used for feature engineering, machine learning, and data visualization based on the obtained radiomic features with these packages: Matplotlib, Numpy, Sklearn, Pandas, Seaborn, and Scipy.

#### Feature Selection

Firstly, the data were randomly divided into training sets and test sets (8:2). Then, a *t*-test was applied to select significantly different features (*P* < 0.05) between the stage I and non-stage I disease in the train set. Then, Least Absolute Shrinkage and Selection Operator (LASSO) regression was used to screen the optimal feature subset.

#### Dataset Balancing

The proportion of stage I and non-stage I group in the training data was imbalanced (38:56). So, the Synthetic Minority Over-Sampling Technique (SMOTE), a kind of oversampling technique, was used to introduce artificial minority instances to even the class distribution before classification. The SMOTE training data was applied to build the predictive model.

#### Classification and Validation

Models were established using a machine learning method of Support Vector Machine (SVM) with the selected radiomic features. Stratified 10-fold cross-validation was carried out to evaluate the generalization ability of the model, which would further split the training data into new training and test sets and use the new training sets to fit the model, while the corresponding new test sets to verify the model for 10 times. Accuracy, sensitivity, specificity, and area under the ROC curve (AUC) were utilized to assess the performance of the model for WT staging in the training cohort and test cohort.

The Mann-Whitney U test was used to compare the differences in age between the stage I group and the non-stage I, and the Chi-Square test was utilized to assess the gender differences. *P* < 0.05 was considered statistically significant. Statistical analysis was carried out by Python.

## Results

### Demographic Data and Clinical Factors

A total of 48 patiens with WT with stage I (22 males and 26 females) disease and 70 non-stage I (36 males and 34 females) were included in the study (Table 1). The median age of patients with WT with both stage I and non-stage I disease was 2.1 years. There was no significant difference in age and sex between the two groups (*P* > 0.05). Clinical factors were also classified. Metastasis and tumor thrombus were significantly different between the stage I and non-stage I groups, whereas anaplastic status was not (Table 1).

**Table 1:** Demographic data and clinical factors of WT patients.

		**WT staging**		***P*-value**
**Variables**		**Stage I**	**Non-Stage I**	
*n*		48	70	
Sex, *n* (%)	**F**	26 (54.2)	34 (48.6)	0.682
	**M**	22 (45.8)	36 (51.4)	
Age, Y (median)		2.1	2.1	0.306
Volume, cm^3^ (median)		287.6	325.2	0.547
Metastasis, *n* (%)	**No**	48 (100.0)	10 (14.3)	<0.001
	**Yes**	0 (0.0)	60 (85.7)	
Tumor thrombus, *n* (%)	**No**	48 (100.0)	49 (70.0)	<0.001
	**Yes**	0 (0.0)	21 (30.0)	
Anaplastic WT, *n* (%)	**No**	48 (100.0)	67 (95.7)	0.270
	**Yes**	0 (0.0)	3 (4.3)	

### Intra- and Inter-observer Reproducibility

The features extraction achieved satisfactory inter- and intra-observer reproducibility. The intra-observer ICC ranged from 0.819 to 1 based on radiologist 1's twice feature extraction. The inter-observer ICCs derived using radiologist 1's first-generated features and radiologist 2's features ranged from 0.726 to 1.

Accordingly, the whole results were based on the features obtained by radiologist 1.

### Feature Extraction and Selection

**Figure 2:**
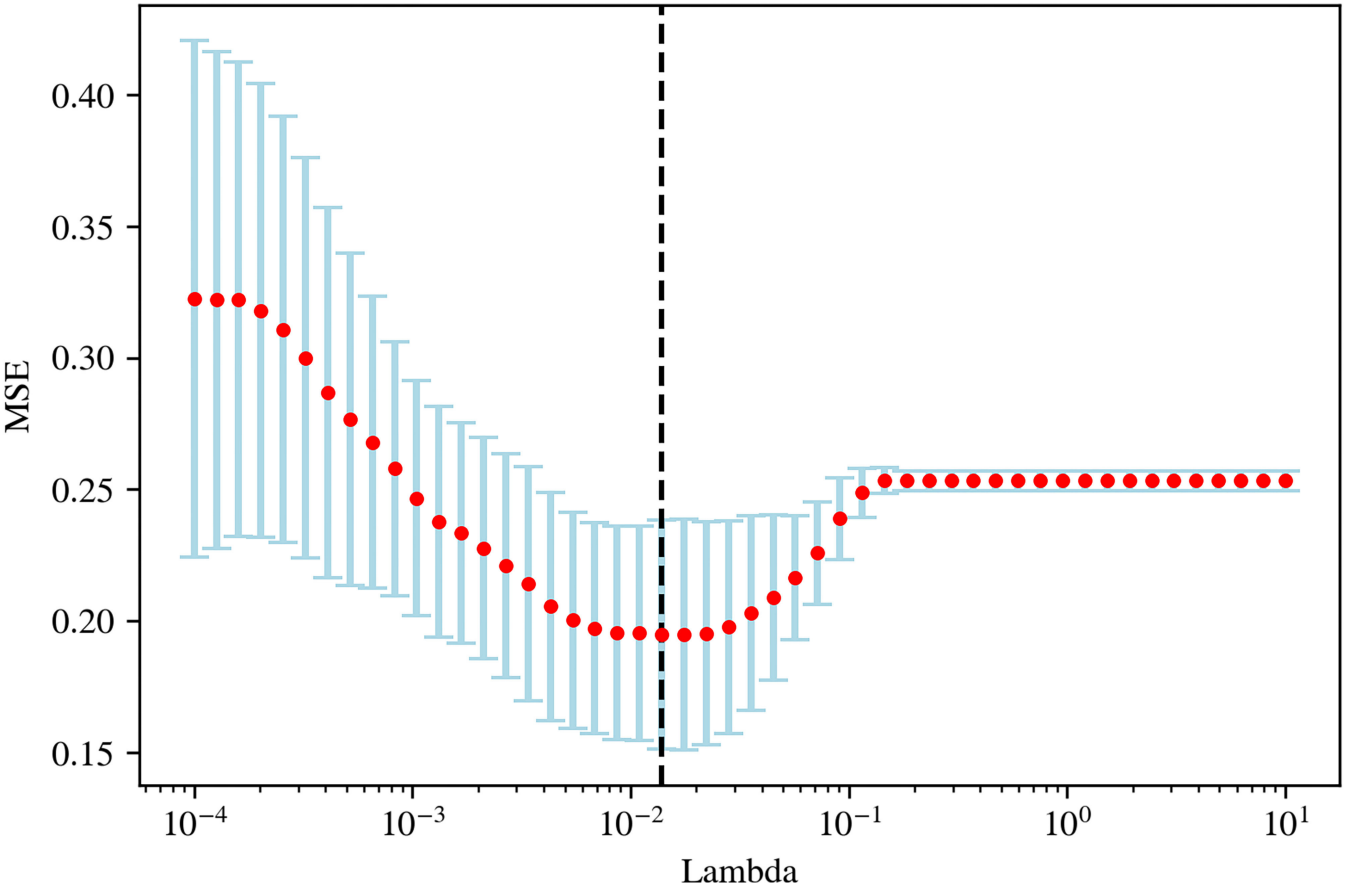
The optimal lambda value (0.0136) in least absolute shrinkage and selection operator (LASSO) regression.

**Figure 3:**
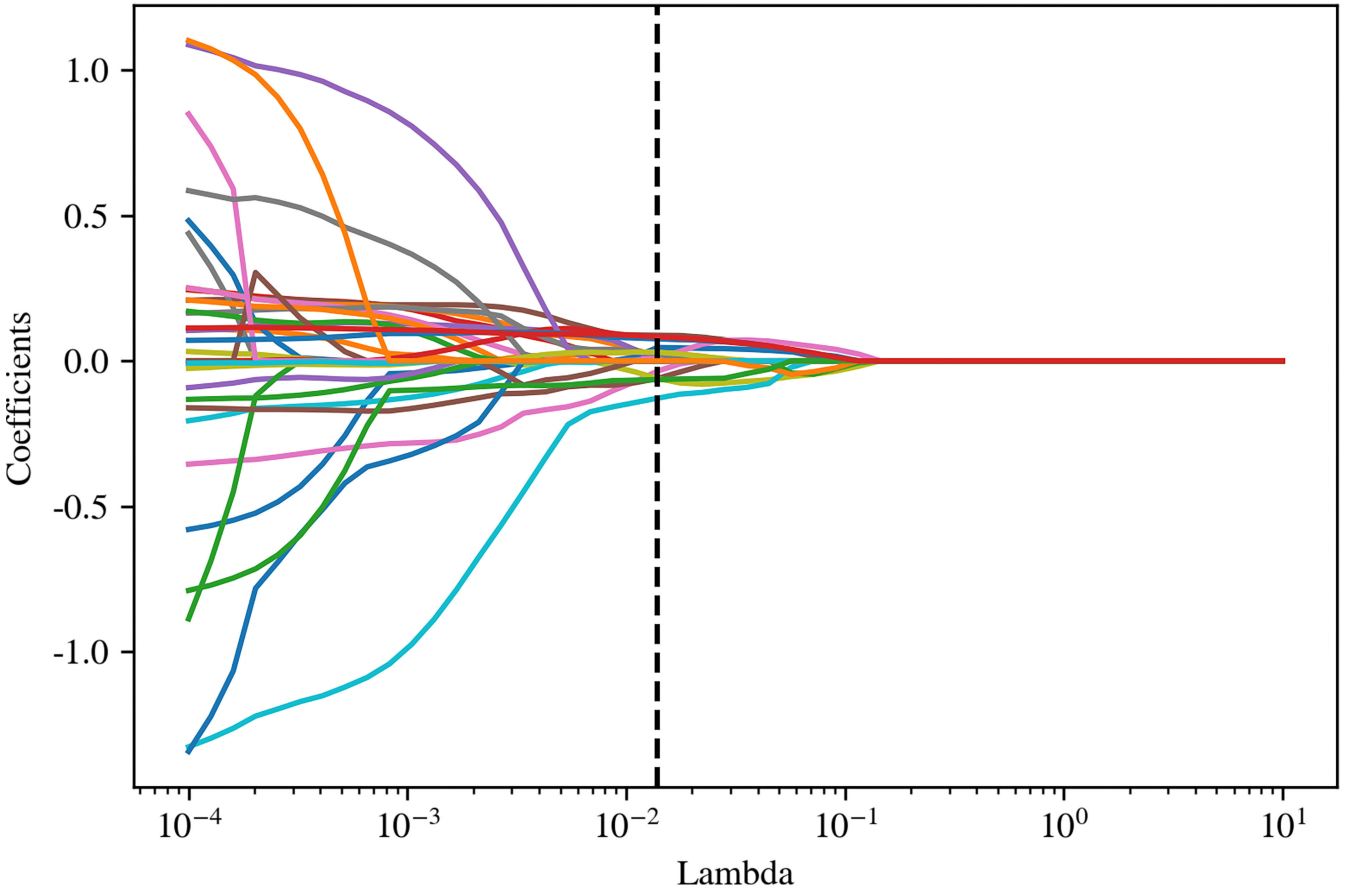
Coefficient profile plot showed the coefficient paths following different lambda values.

Pyradiomics was used to extract radiomic features from original and filtered images, which had been preprocessed with resampling and normalization, yielding 1,781 features for each case. Subsequently, a *t*-test, a kind of univariate feature selection, was applied and 35 features were selected, which were narrowed down to 15 by further using the Lasso algorithm with the most proper lambda value of 0.0136 (Figures 2, 3).

**Figure 4:**
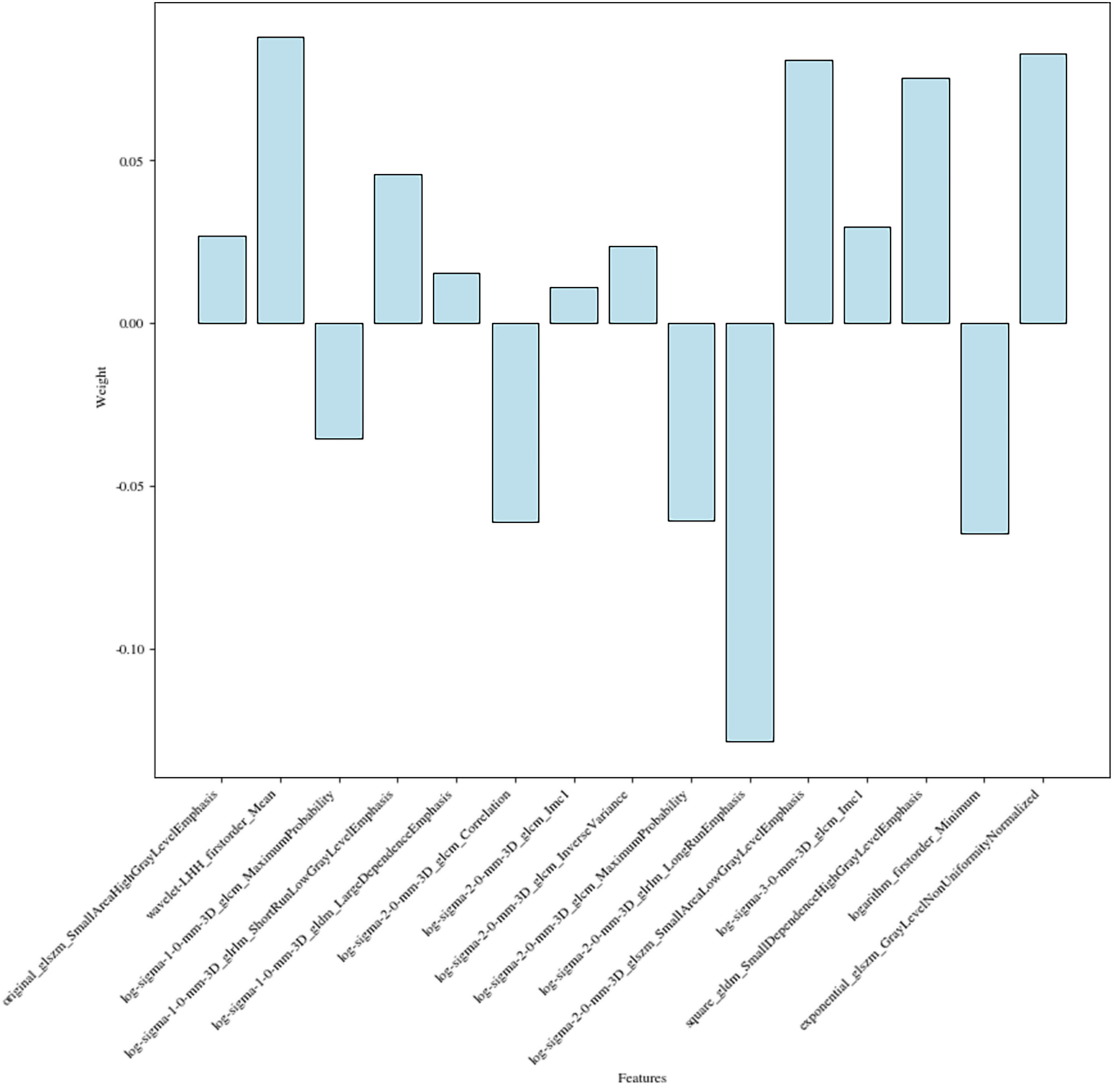
Radiomic features related to WT staging and their weights.

The 15 radiomics features had different weights in distinguishing WT stages (Figure 4).

The presence of collinearity may lead to serious problems in the model, and 0.8 and 0.9 are commonly used cut-offs (19), indicating a strong linear correlation. So, the correlation of the selected features was analyzed and a correlogram (Supplementary Figure S1) was plotted to show that no multicollinearity existed according to the given cut-offs.

Finally, a total of 15 features were used for model fitting, including 2 first-order features and 13 texture features (Supplementary Table S2).

### Machine Learning Results and Model Validation

Approximately 80% of the patients with WT were randomly chosen as the training set, with the remaining set as the test set.

**Figure 5:**
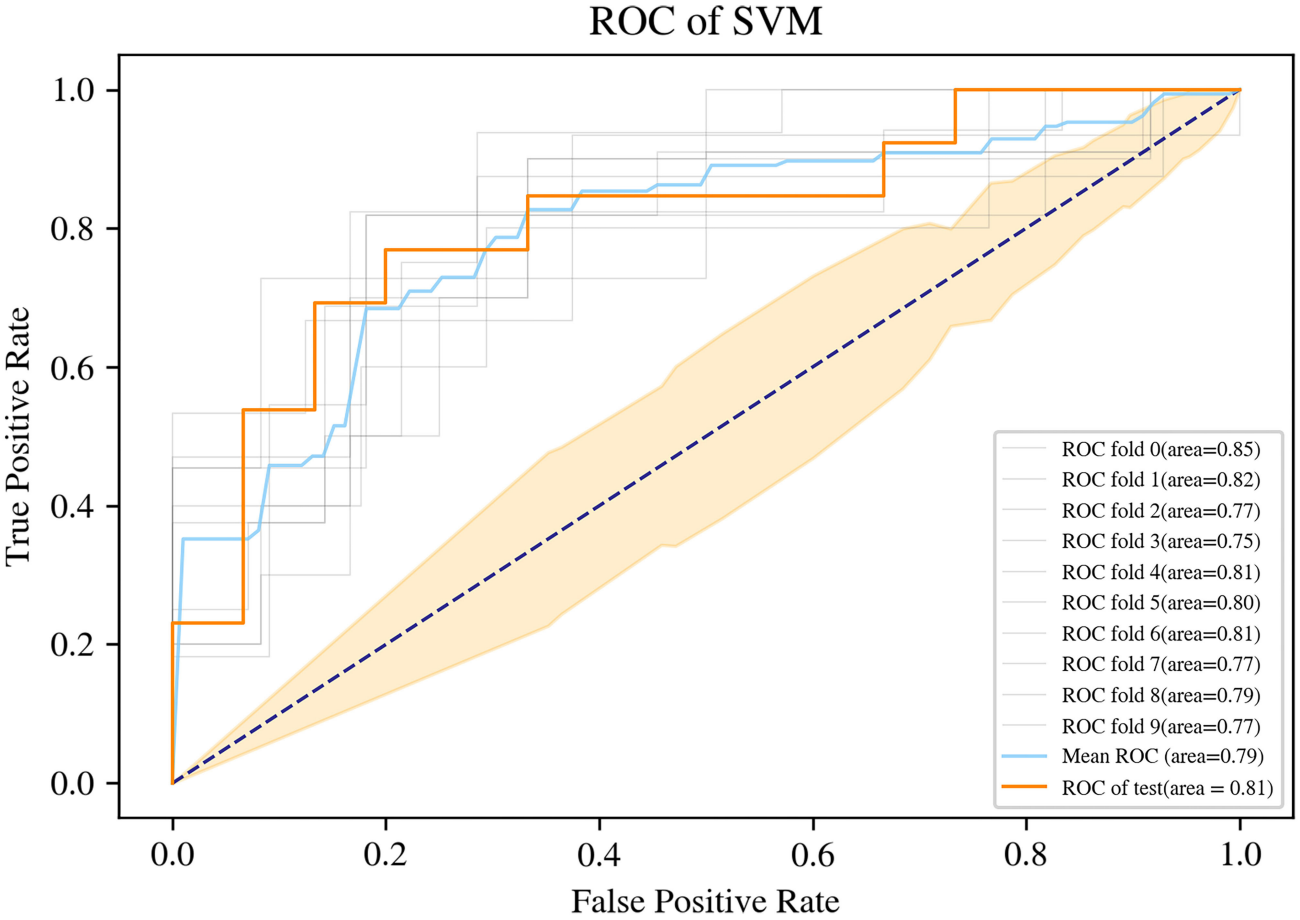
Receiver operating characteristic (ROC) curve of the support vector machine (SVM) machine learning method.

The SVM for WT staging based on selected radiomic features was fitted, achieving a mean accuracy of 0.75, a mean AUC of 0.79 (95% CI, 0.773–0.815) with a coefficient of variation of 3.76% in 10-fold cross-validation, and a mean sensitivity/specificity of 0.71/0.80. On the independent test dataset, the model reached 0.79 on the accuracy, 0.81 on AUC, 0.87 on sensitivity, and 0.69 on specificity (Figure 5). The findings of the machine-learning model of SVM for classifying stage I vs. non-stage I disease were seen in Table 2.

**Table 2:** The SVM model's performance in training set and test set.

	**AUC**	**Sensitivity**	**Specificity**	**Accuracy**
Training set (mean, 95% CI)	0.79	0.71	0.80	0.75
	0.773–0.815	0.646–0.782	0.734–0.868	0.705–0.788
Testing set	0.81	0.87	0.69	0.79

## Discussion

We investigated the capacity of radiomics combined with the machine-learning method to identify the stage of WT in pediatric patients prior to surgery. The fifteen-feature–based SVM machine learning model was trained to be effective for WT staging, which could classify patients with WT into stage I class and non-stage I class, with an AUC of 0.81, the accuracy of 0.79, the sensitivity of 0.87, and specificity of 0.69, leading the potential to serve as non-invasive WT staging prior to surgery.

To our knowledge, no other teams have studied preoperative non-invasive WT staging methods. The radiomics-based SVM can benefit patients with WT in two ways. Firstly, it enables a more rational design of surgical protocols, such as choosing nephron-sparing surgery (NSS), because potential cases for NSS should have low-stage tumors; evidence suggests that prognosis for anaplastic (high-risk) and other subtypes (intermediate and low-risk) is similar in patients with stage I disease (20). Secondly, it has the potential to facilitate the SIOP system in developing more personalized preoperative chemotherapy regimens; patients in stage I have the opportunity to receive less chemotherapy to reduce treatment-related toxicity and late side effects while maintaining efficacy due to the significant differences in metastasis and tumor thrombosis between the stage I and non-stage I groups (21).

Previous studies have directly associated radiomic features with genomic, transcriptomic, and proteomic characteristics (22) and believed that radiomics techniques could evaluate both molecular and clinical traits of a tumor in a non-invasive way relying on the features extracted from medical images. We collected 1,781 features from CT images of each patient, then, utilized a feature selection procedure to filter non-redundant, robust, and informative features for WT staging, and finally screened out 15 potential predictors (2 First-order and 13 Second-order statistics) for building a supervised machine learning model.

These two First-order statistics were Mean and Minimum, which described the average and the lowest gray level intensity within the image region as defined by the mask—representing the density of the tumor—and were applied to quantify tumor phenotypic features (23). High stages, massive tumors, and liquefied necrotic regions were usually correlated (24) in most malignant neoplasms. In other words, higher stages meant more necrotic areas and less homogeneous.

The other 13 potential predictors from GLCM, GLDM, GLRLM, and GLSZM classes were all second-order statistics, also known as texture features, which depicted differences in texture based on gray tone spatial dependencies (23) and had a higher weight than the screened first-order statistics.

Texture analysis has been suggested as a way to discriminate malignant masses from benign ones and to predict treatment response and prognosis (25) because of its relationship with intratumoral heterogeneity. For example, the maximum probability of GLCM described the most frequently occurring texture features in the image within the ROI, and a lower probability suggested a more complicated texture pattern, which was consistent with this study—the maximum probability value was lower in non-stage I group.

There were some shortcomings in the current study. Given that tumor thrombi and hematogenous metastases (stage III and IV) would be visible on the diagnostic CT, the current radiomics study focused mainly on the mass of WT, and lacked inclusion of lymph nodes in the segmentation, which might result in a limited predictive power of our radiomics model. Moreover, due to the limitations of small sample size and sample heterogeneity, retrospective analysis, and single-center design, there may be a selection bias that limits the robustness and generalization ability of our radiomics model. Further studies in a larger prospective cohort and independent test datasets from other centers are needed in the future.

## Conclusions

In conclusion, there were CT-based radiomic features that were independently associated with WT stage I disease in pediatric patients, and the SVM machine learning model built with them could act as an effective and non-invasive technique to differentiate stage I and non-stage I WT preoperatively.

## Data Availability Statement

The raw data supporting the conclusions of this article will be made available by the authors, without undue reservation.

## Ethics Statement

The studies involving human participants were reviewed and approved by the Institutional Review Board of the Children's Hospital, Zhejiang University School of Medicine. Written informed consent from the participants' legal guardian/next of kin was not required to participate in this study in accordance with the national legislation and the institutional requirements.

## Author Contributions

X-HM and LS contributed equally to this work in designing this project, analyzing data, and writing this manuscript. J-WL, Y-SD, H-CZ, and MH mainly the inclusion, exclusion of cases, and acquisition of CT images. H-CZ and XJ delineated the area of interest in our medical images, and T-TL was primarily responsible for the development of machine learning. QS supervised and guided the development of this project and the writing of this article. X-HM takes mainly responsibility for the integrity of the content of the paper and each author have participated sufficiently in the submission to take public responsibility for the content. All authors contributed to the article and approved the submitted version.

## Funding

This research was funded by the Medical Health Science and Technology Project of Zhejiang Province (project number: 2022492239).

## Conflict of Interest

The authors declare that the research was conducted in the absence of any commercial or financial relationships that could be construed as a potential conflict of interest.

## Publisher's Note

All claims expressed in this article are solely those of the authors and do not necessarily represent those of their affiliated organizations, or those of the publisher, the editors and the reviewers. Any product that may be evaluated in this article, or claim that may be made by its manufacturer, is not guaranteed or endorsed by the publisher.
