# Supplementary material for: Machine Learning-Based CT Radiomics Method for Identifying the Stage of Wilms Tumor in Children
Source: Front Pediatr. 2022 May 23;10:873035. doi: 10.3389/fped.2022.873035 (PMC9168275; doi:10.3389/fped.2022.873035)
Supplement: Supplementary file 1 [file Data_Sheet_1.docx]

# Supplementary material

Table S1. Stages of 118 WT patients recruited

|  | Stage I | Non-stage I | | | Total |
| --- | --- | --- | --- | --- | --- |
|  |  | Stage II | Stage III | Stage IV |  |
| WT cases | 48 | 43 | 18 | 9 | 118 |
| Total | 48 | 70 | | |  |


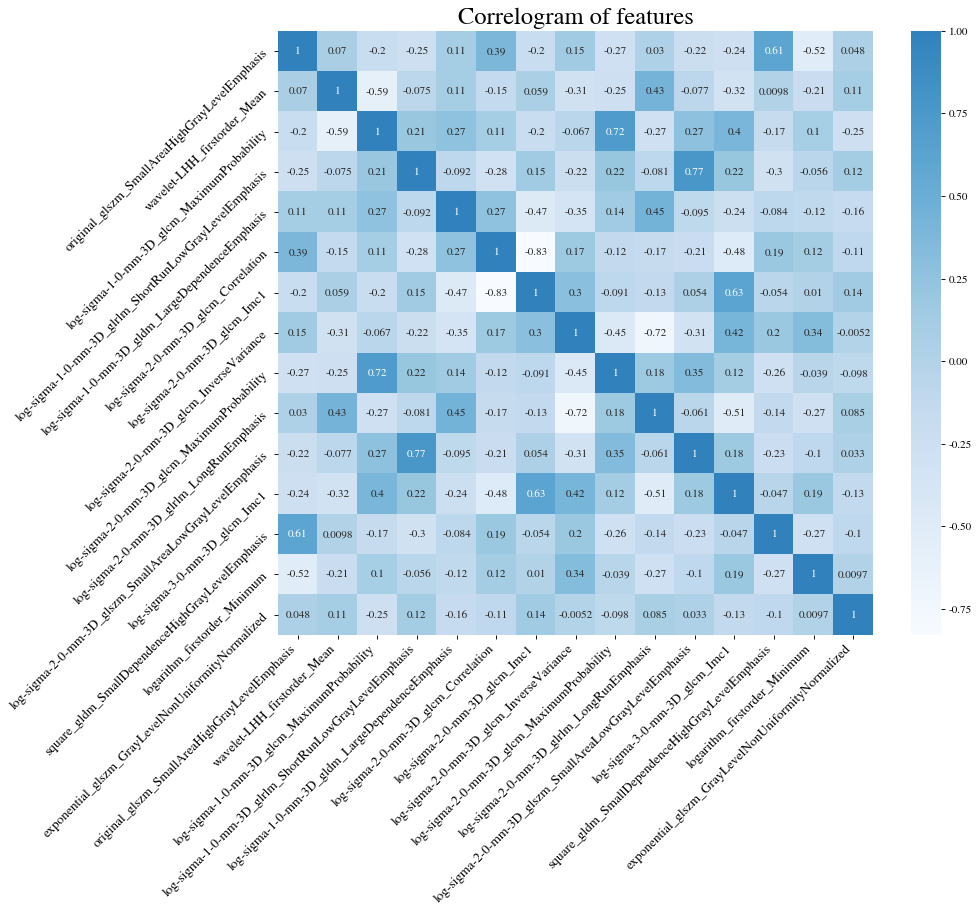


Fig. S1 Fifteen correlation coefficient heat map of radiomic features

Table S2 Fifteen features selected for machine learning along with their weights

| **Selected Features** | | | **Weight** |
| --- | --- | --- | --- |
| **Filters** | **Classes** | **Features** |  |
| wavelet | Firstorder | Mean | 0.087998 |
| logarithm | Firstorder | Minimum | -0.06448 |
| LoG | GLCM | MaximumProbability | -0.03537 |
| LoG | GLCM | Correlation | -0.06112 |
| LoG | GLCM | Imc1 | 0.011275 |
| LoG | GLCM | InverseVariance | 0.023932 |
| LoG | GLCM | MaximumProbability | -0.06053 |
| LoG | GLCM | Imc1 | 0.029728 |
| LoG | GLDM | LargeDependenceEmphasis | 0.015652 |
| square | GLDM | SmallDependenceHighGrayLevelEmphasis | 0.075376 |
| LoG | GLRLM | ShortRunLowGrayLevelEmphasis | 0.045931 |
| LoG | GLRLM | LongRunEmphasis | -0.12846 |
| LoG | GLSZM | SmallAreaLowGrayLevelEmphasis | 0.080763 |
| original | GLSZM | SmallAreaHighGrayLevelEmphasis | 0.026901 |
| exponential | GLSZM | GrayLevelNonUniformityNormalized | 0.082749 |
